# The Road to Transfusion-free Craniosynostosis Repair in Children Less Than 24 Months Old: A Quality Improvement Initiative

**DOI:** 10.1097/pq9.0000000000000331

**Published:** 2020-07-10

**Authors:** Amy B. Beethe, Rachel A. Spitznagel, Jane A. Kugler, Jessica K. Goeller, Marcellene H. Franzen, Ryan J. Hamlin, Thomas J. Lockhart, Elizabeth R. Lyden, Kimberly R. Glogowski, Michelle M. LeRiger

**Affiliations:** From the * Department of Anesthesiology, University of Nebraska Medical Center, Omaha, Neb.; † Division of Pediatric Anesthesiology, Children’s Hospital & Medical Center, Omaha, Neb.; ‡ Department of Anesthesiology, Boystown National Research Hospital, Omaha, Neb.; § Department of Biostatistics, University of Nebraska Medical Center, Omaha, Neb.; ¶ Department of Clinical Perfusion, Children’s Hospital and Medical Center, Omaha, Neb.

## Abstract

**Introduction:**

Pediatric craniofacial reconstruction has historically resulted in extensive blood loss necessitating transfusion. This single-center quality improvement initiative evaluates the impact of perioperative practice changes on the allogeneic transfusion rate for children 24 months and younger of age undergoing craniofacial reconstruction.

**Methods:**

At project initiation, an appointed core group of anesthesiologists provided all intraoperative anesthetic care for patients undergoing craniofacial reconstruction. Standardized anesthetic guidelines established consistency between providers. Using the Plan-do-check-act methodology, practice changes had been implemented and studied over a 5-year period. Improvement initiatives included developing a temperature-management protocol, using a postoperative transfusion protocol, administering intraoperative tranexamic acid, and a preincisional injection of 0.25% lidocaine with epinephrine. For each year of the project, we acquired data for intraoperative and postoperative allogeneic transfusion rates.

**Results:**

A cohort of 119 pediatric patients, ages 4–24 months, underwent anterior or posterior vault reconstruction for craniosynostosis at a tertiary children’s hospital between March 2013 and November 2018. Intraoperative and postoperative transfusion of allogeneic blood products in this cohort decreased from 100% preintervention to 22.7% postintervention.

**Conclusions:**

Interdepartmental collaboration and practice modifications using sequential Plan-do-check-act cycles resulted in a bundle of care that leads to a sustainable decrease in the rate of intraoperative and postoperative allogeneic blood transfusions in patients less than 24 months of age undergoing craniosynostosis repair. This bundle decreases the risk of transfusion-related morbidity for these patients. Other institutions looking to achieve similar outcomes can implement this project.

## INTRODUCTION

Craniosynostosis is a condition in which one or more sutures close prematurely. The incidence is approximately 1:1,800 births.^1^ Surgical repair is usually performed in the first year of life to avoid increased intracranial pressure and consequent developmental delay, as well as to improve cosmetic outcomes. The operation is associated with substantial perioperative bleeding, necessitating transfusion of blood products.

The Pediatric Craniofacial Collaborative Group (PCCG) analyzed 935 children less than or equal to 24 months of age with craniosynostosis and reported that the likelihood of a perioperative transfusion was 95%. In this study, the mean volume of erythrocyte containing blood products transfused intraoperatively for infants was 37.3 ± 26.2 ml/kg, and the mean volume transfused postoperatively was 16.7 ± 9.3 ml/kg.^2^

Stricker et al^2^ suggested that surgical technique may be the most significant determinant of blood loss and the need for transfusion. He proposed that those institutions with below-average transfusion rates could establish themselves as centers where perioperative transfusion is the outlier rather than the norm by adopting multimodal blood management practices. Many studies have investigated strategies to reduce blood loss and transfusion in children undergoing craniofacial reconstruction.^3,4^ The use of postoperative transfusion guidelines showed a 60% decrease in postoperative transfusion, mainly through the use of less fresh frozen plasma.^5^ Dadure et al^6^ studied the use of intraoperative tranexamic acid in children undergoing craniosynostosis surgery. They found a reduction in the volume of packed erythrocytes transfused intraoperatively by 85% and perioperatively by 57% versus placebo. Goobie et al^7–9^ confirmed the efficacy, safety profile, and pharmacodynamics of tranexamic acid in craniosynostosis surgery. Vega et al^3^ established that the use of a perioperative protocol (preoperative erythropoietin/iron therapy, intraoperative blood recycling, and acceptance of a lower level of hemoglobin before transfusion) resulted in a 40% reduction in the number of patients requiring intraoperative transfusions and shorter hospitalization stays (3.4 versus 2.6 days). None of these single interventions led to reports of transfusion-free hospitalizations.

Therefore, with below-average operative times, our institution felt that it was realistic to achieve transfusion-free craniosynostosis repair and initiated a performance improvement project with means of tracking transfusion rates over time. Improvement initiatives included developing a temperature-management protocol, using a postoperative transfusion protocol, administering intraoperative tranexamic acid, and preincisional injection of 0.25% lidocaine with epinephrine. This study’s primary objective was to assess the effect of these interventions on total allogeneic blood transfusion rates in children less than 24 months of age undergoing craniosynostosis repair.

## METHODS

The Pediatric Institutional Review Board at the University of Nebraska Medical Center approved the study. This article adheres to the applicable SQUIRE (Standards for Quality Improvement Reporting Excellence) 2.0 guidelines.^10^ The time frame of this study was from March 2013 to November 2018. After the procedure, parents gave written consent to allow the addition of their child’s surgical data to the PCCG database. As a quality improvement initiative, no ethical dilemmas ensued when instituting the described interventions. The primary outcome of interest was the intraoperative, postoperative, and total allogeneic transfusion rates for children less than 24 months of age undergoing craniofacial reconstruction. The data was analyzed to evaluate for a statistically significant difference in allogeneic blood transfusion rates between the baseline data of 2013 and the final year data of the project in 2018.

The craniofacial team, comprising one craniofacial surgeon, consistent pediatric neurosurgeons, and a newly developed core group of anesthesiologists, created recommendations for perioperative management. Written anesthetic guidelines for craniofacial reconstruction procedures were developed and followed by this subgroup of anesthesiologists. Practice changes were implemented and studied over a 5-year period. For this project, the PCCG database was examined only at institutional transfusion rates before and after the implementation of process-improvement initiatives. The outcome was the percentage of patients undergoing cranial vault reconstruction requiring an allogeneic blood transfusion.

### Anesthetic Management

The anesthetic technique consisted of an inhalational induction using sevoflurane, followed by peripheral intravenous catheter placement. Patients received a neuromuscular blocking agent, followed by intubation, and securement of the endotracheal tube. An additional intravenous catheter, an arterial line, and an indwelling urinary catheter with temperature-monitoring capabilities were placed after turning the bed 90 degrees. Before incision, a 20 ml/kg bolus of crystalloid was administered. The early turning of the bed allowed surgeons to immediately start preoperative surgical preparation, which eventually included the administration of up to 20 ml of 0.25% lidocaine with 1:400,000 epinephrine at the incision site, allowing for the maximal hemostatic effect of the epinephrine. Surgeons, anesthesiologists, and nurses worked concurrently. An intraoperative transfusion protocol had not been established. The decision to transfuse relied on clinical bleeding assessment, patient hemodynamics, review of laboratory values, and discussion with the surgeons. Maintenance of mean arterial blood pressure was in an age-appropriate range. Total crystalloid administration ranged between 30 and 100 ml/kg. Following the operation, patients were extubated and cared for in the pediatric intensive care unit.

### Intraoperative Blood Salvage

Cell saver was used for all patients, including for patients in 2013. The Fresenius Kabi C.A.T.S plus Continuous Auto Transfusion System (Terumo Cardiovascular, Ann Arbor, Mich.) processed the shed blood from the surgical field during all craniofacial procedures for retransfusion. As little as 25 ml could be returned to the patient for retransfusion. The “High-Quality Wash” program was used for processing the red blood cells for retransfusion. Following the American Association of Blood Bank guidelines, autotransfusion-prepared blood products expired 8 hours after processing.

Surgeons were diligent in controlling hemostasis during dissection, using cautery and bone wax. Throughout the entire dissection and reconstruction, surgical technologists and surgeons captured the majority of blood loss with the cell saver. We placed an irrigation pouch under the cranium, and then throughout the case, suctioning and processing the blood in the pouch took place by way of the cell saver. The surgical technologists put blood-soaked lap pads in saline to soak and then processed that solution via the cell saver.

### Plan-do-check-act Cycles

Using plan-do-check-act (PDCA) methodology, a multidisciplinary team implemented processes of improvement in the perioperative care of children of age 24 months and younger undergoing craniofacial reconstruction with the primary aim of decreasing the overall perioperative allogeneic transfusion rate.^11^ Improvement initiatives included the development of a temperature-management protocol, utilization of a postoperative transfusion protocol, administration of intraoperative tranexamic acid, and preincisional injection of 0.25% lidocaine with epinephrine.

#### Perioperative Temperature Management

After initial data analysis for 2013, there was concern regarding prolonged periods of perioperative hypothermia. Patients were regularly presenting from the preoperative unit hypothermic. We set the operating room temperatures too low, and the method of temperature monitoring was inconsistent. In 2014, a preoperative warming algorithm was developed and instituted as one of the first PDCA cycles. With the goal of warming patients to 37.0–37.6°C, forced air warmers or warm blankets were used based on patient tolerance at least 30 minutes before the scheduled surgical time. All patients received an indwelling urinary catheter to monitor the intraoperative temperature. Twenty-two degrees Celsius became the lowest temperature allowed in the operating room, and forced air warmers and intravenous fluid warmers became the standard practice.

#### Postoperative Transfusion Protocol

The implementation of a postoperative transfusion protocol occurred at approximately the same time. The protocol stated that if a patient had hemoglobin less than 7 g/dl and was hemodynamically stable, then a surgical consultation was required before initiation of blood products. This protocol was imperative because, at the time of implementation, there were inconsistencies in the transfusion threshold in the pediatric intensive care unit.

#### Antifibrinolytic Therapy

In 2015, we instituted the use of tranexamic acid intraoperatively. Upon initiation, dissemination of information occurred via verbal communication. After consideration of institutional operative times and data published on the pharmacodynamics of tranexamic acid, a tranexamic bolus of 12.5 mg/kg before incision became the new standard.^9^ Education was enhanced via a departmental journal club reviewing articles on the safety and efficacy of tranexamic in craniofacial procedures to promote provider compliance. We updated the written anesthetic guidelines to provide further clarification for the department.

#### Preincisional Injection of 0.25% Lidocaine with Epinephrine

In 2016, we instituted the injection of up to 20 ml of 0.25% lidocaine with 1:400,000 epinephrine before surgical incision.

### Statistical Analysis

To assess outcomes, the PCCG database was examined for the institution’s data. The query yielded 119 patients less than or equal to 24 months of age who underwent anterior or posterior vault reconstruction for craniosynostosis between March 2013 and November 2018. Transfusion data were organized by year and then analyzed by dichotomized transfused yes or no—categorical data used counts and percentages. Intraoperative, postoperative, and total transfusion evaluation occurred from the data. The utilization of dichotomized transfusion variables allowed usage of the Fisher’s exact test to compare intervention groups. Data from 2013 were considered the baseline data, and it formed the initial dataset to compare with the data from 2018. To ensure data accuracy, we conducted random audits. A *P* value <0.05 was considered statistically significant.

## RESULTS

**Fig. 1.**
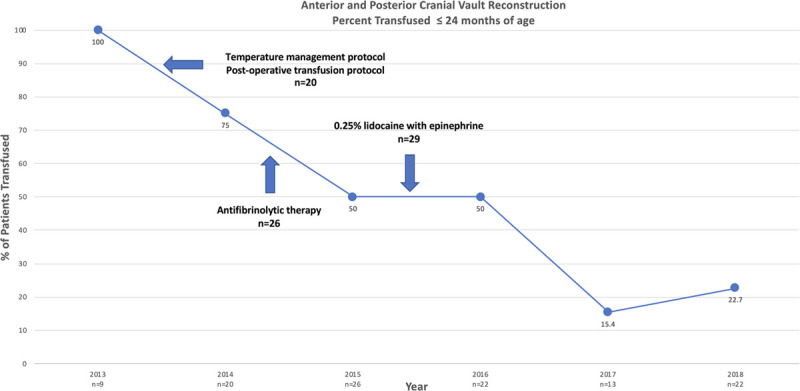
Autologous transfusion rates for children less than 24 months of age undergoing anterior and posterior cranial vault reconstruction throughout the quality improvement initiative. This line chart displays the transfusion rate over time from 2013 to 2018 with the PDCA cycles shown.

As seen in Figure 1, the institution’s total allogeneic transfusion rate for this patient cohort started at 100%. After each intervention, documentation of improvement occurred, with a final overall allogeneic transfusion rate of 22.7%. Table 1 summarizes the intraoperative, postoperative, and total allogeneic transfusion rates by year. Data analysis between 2013 and 2018 demonstrated a statistically significant association between intraoperative, postoperative, and total allogeneic transfusion rates. Patient age, total hospital length of stay, operative times (incision to closure), preoperative hemoglobin, lowest intraoperative hemoglobin, and lowest postoperative hemoglobin are summarized by year in Table 2.

**Table 1.**
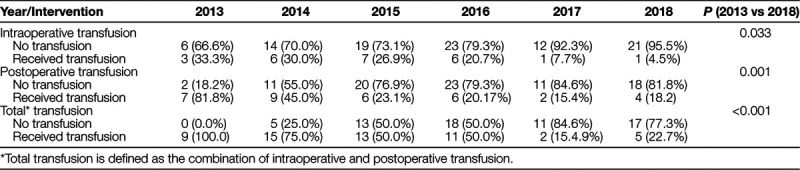
Comparison of Allogeneic Transfusion Rates by Intervention Year

**Table 2.**
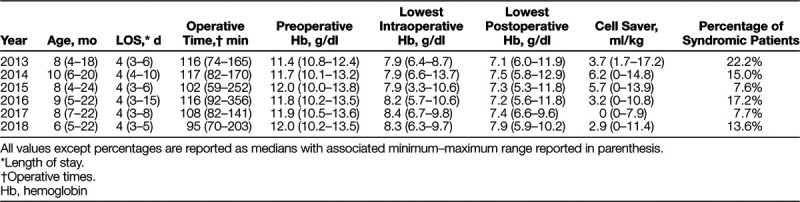
Patient Demographics, Length of Stay, and Perioperative Hemoglobin Values

## DISCUSSION

This project used the PDCA methodology.^11^ At project initiation, a subset of core anesthesiologists performed all craniofacial procedures, which allowed for more efficient and consistent adherence to the new standard practice guidelines created. This intervention also ensured that each team member performed an adequate number of cases to maintain proficiency. The multidisciplinary approach allowed for the expansion of interventions to include preoperative and postoperative care. These multidisciplinary practice changes were associated with a 77.3% reduction in the total allogeneic transfusion rate in patients less than 24 months of age undergoing craniofacial reconstruction from 2013 to 2018 (Fig. 1). Our 2018 transfusion rate of 22.7% is comparable to the transfusion rate of 26% found by Thompson et al^12^ in their review of endoscopic craniosynostosis repair, which is associated with significantly less blood loss than open cranial vault repair. The PCCG reported a multicenter observational study of a perioperative transfusion rate of 95% in children less 24 months of age undergoing craniosynostosis repair.^2^

The same surgeons used the same operative techniques throughout the study. The median operative times remained relatively consistent (Table 2), and there were no changes to the anesthetic technique other than those implemented as part of the project. Therefore, we feel these factors did not account for any change in the transfusion rate identified. Older patients are traditionally at a lower risk of requiring a transfusion simply due to blood volume associated with the patient’s size. So, while changes in patient demographics could undoubtedly account for reductions in transfusion rates, all of our patients were less than 24 months of age, and the median age of our patients remained relatively consistent over the study duration (Table 2). Additionally, the percentage of syndromic patients was relatively constant (Table 2).

Inherent weaknesses secondary to the retrospective nature of the study warrant further discussion. Providers may have developed a bias toward tolerance of a lower intraoperative or postoperative hemoglobin level following implementation, impacting the decision on whether or not to transfuse. Although acceptance of a lower intraoperative transfusion trigger has shown to decrease transfusion rates for cranial vault remodeling, we did not use a numerical intraoperative transfusion trigger.^3^ Instead, the decision for an intraoperative allogeneic transfusion relied on the amount of clinical bleeding, the patient’s hemodynamic status and laboratory values, and a team discussion between the anesthesiologist and surgeons. Permissive hypotension was not a practiced modality for decreasing blood loss. Table 3 provides details of all intraoperative and postoperative complications. The table records explicitly any patient with hypotension necessitating vasopressor administration due to inadequate hemodynamic response to fluid resuscitation. There was no recommended vasoactive agent of choice; rather, this was at the discretion of the anesthesiologist. Table 2 displays the lowest intraoperative hemoglobin levels and demonstrates that permissive anemia was not an accepted practice. The median lowest intraoperative hemoglobin level ranged from 7.9 to 8.4 g/dl. One outlier noted in 2015 reported an intraoperative hemoglobin value of 3.3 g/dl. Upon chart review, due to severe hematuria and cardiovascular instability during a blood transfusion, the anesthesiologist stopped the transfusion due to concern for a possible transfusion reaction. However, due to active bleeding without compatible blood products, the hemoglobin level decreased to 3.3 g/dl before the compatible blood products were available. With the removal of this outlier, the minimum intraoperative hemoglobin value for 2015 would be 6.3 g/dl. The lowest median postoperative hemoglobin level ranged from 7.1 to 7.9 g/dl. The postoperative transfusion protocol emphasized communication and joint decision making between the pediatric intensive care unit and the surgical team when the hemoglobin fell below 7 g/dl, and the patient was hemodynamically stable. Because this protocol was very explicit, it is unlikely that bias contributed to the decision to transfuse.

**Table 3.**
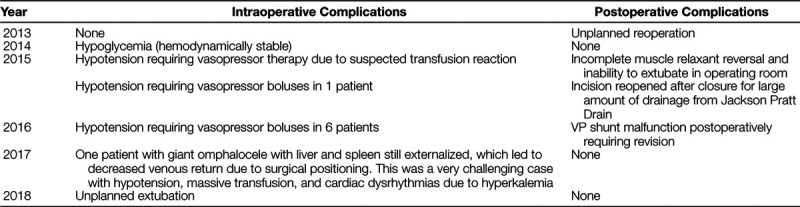
Intraoperative and Postoperative Complications by Year

Throughout the study, the cell saver was used for all the cases. If blood loss was substantial enough to obtain cell saver volume, then the autologous blood was returned to the patient. In this study, the accounting of the cell saver volumes transfused took place in ml/kg. This practice is supported by Krajewski et al,^13^ who demonstrated that cell salvage avoided allogeneic blood transfusion in 31% of patients undergoing craniosynostosis correction. In 2017, the median autologous volume transfused was 0 ml/kg. The surgical technique involved and the cell saver capability explain this result. Traditionally, with the exposure of the child’s head during craniofacial repair, there is not a focal point of bleeding but rather a generalized ooze throughout the exposed cranium, making it difficult to recover the minimal blood loss volume required to produce a cell salvage product. Therefore, as blood loss necessitating allogeneic blood transfusion decreased over the years, so did the ability to capture enough blood loss for cell salvage processing.

Not all providers immediately adopted tranexamic acid into their practice despite incorporating tranexamic acid into the intraoperative guidelines. The entire team did not use tranexamic acid until the year 2017. Because of the relatively low transfusion rate at the institution, some providers felt this additional measure was unnecessary. As further data on the subject became available, late adopters were willing to implement the change, highlighting the importance of data-driven or evidence-based changes. Improved communication regarding the change in guidelines with supportive data may have hastened compliance, resulting in earlier adaptation. Based on this experience, communication should occur in multiple formats and on numerous occasions.

Preoperative iron supplementation was a confounding variable of our study. Iron deficiency is the leading cause of preoperative anemia, and preoperative iron supplementation has been suggested as a method to mitigate this.^14^ Although there is insufficient evidence to support the routine use of iron therapy before major surgery, iron therapy’s preoperative initiation showed an increase in hemoglobin levels.^14–16^ In 2016, surgeons prescribed supplemental iron at the patient’s preoperative clinic visit before the scheduled operation. Chart review from the day of the patient’s operation revealed whether the parents reported any preoperative iron supplementation. In 50% of records in 2017 and 60% of those in 2018, parents reported taking the prescribed preoperative iron supplementation. In addition to problems with patient compliance, there was poor standardization of type, dose, and timing of the prescription of iron supplementation. Therefore, we did not include it as part of a PDCA cycle. Another notable limitation was the control size of only 9 patients. As with many new surgical programs, the initial number of patients was smaller, and over the years, the program grew substantially. Nonetheless, the control year had noticeably fewer patients than in subsequent years.

The improvement initiatives ultimately resulted in a bundle of care. We feel that these processes for improvement can be implemented by other institutions and other major pediatric surgical operations. With the caveat, the meticulous surgical technique is present as a baseline requirement. Because of the clinical significance of the results, anesthesia providers now trust the importance of using high-quality guidelines to establish consistency and sustainability. They may be more open to adopting additional guidelines in the future.

Further data analysis is warranted as institutional predictions regarding which patients will require blood transfusion based on age, weight, and co-morbidities would allow the perioperative team to better decide if a benefit existed to delaying surgery until the patient was older and/or larger. Implementation of an institutional pediatric blood management program could be the next step in further reducing these patients’ risk of transfusion and is well described in the Australian National Health Authority’s Patient Blood Management Guidelines.^17^ Pediatric blood management programs described by Goobie et al^18^ utilized evidence-based treatment strategies to “screen, diagnose and properly treat anemia, coagulopathies and minimize bleeding, using goal-directed therapy and leverages a patient’s physiologic ability to adapt to anemia while definitive treatment is undertaken.”

The findings of this study are that (1) multidisciplinary team–based surgical care and (2) the use of PDCA cycles to incorporate a bundle of care can lead to reductions of allogeneic blood product transfusion in the total period for craniofacial reconstruction. We have shown that children under 24 months of age can have a transfusion-free hospital course for craniofacial reconstruction. This multimodal approach could be implemented by other institutions or in other major pediatric surgeries. This review is powered by the controlling for the same surgeons, a core group of anesthesiologists, stable operating times, cell saver therapy, and the patient’s age range.

## DISCLOSURE

The authors have no financial interest to declare in relation to the content of this article.

## ACKNOWLEDGMENT

The authors acknowledge the anesthesiology craniofacial team, the surgical team, the perfusionists, the preoperative nurses, and the pediatric intensivists who participated in the care of craniofacial patients throughout the study.
